# External Communication of Autonomous Crews Under Simulation of Interplanetary Missions

**DOI:** 10.3389/fphys.2021.751170

**Published:** 2021-11-09

**Authors:** Natalia Supolkina, Anna Yusupova, Dmitry Shved, Vadim Gushin, Alexandra Savinkina, Svetlana A. Lebedeva, Angelina Chekalina, Polina Kuznetsova

**Affiliations:** ^1^Russian Federation State Scientific Center, Institute of Biomedical Problems of the Russian Academy of Sciences, Moscow, Russia; ^2^Moscow Aviation Institute, National Research University, Moscow, Russia; ^3^Institute of Biomedical Problems, Russian Academy of Sciences (RAS), Moscow, Russia

**Keywords:** space analogs, interplanetary flight, ground-based space simulation, coping strategies, mixed-gender crew, crew communication, SIRIUS, isolation

## Abstract

Two experiments, with 17-day and 120-day isolation, were carried out within the frame of the Scientific International Research in Unique Terrestrial Station (SIRIUS) international project at the Institute of Biomedical Problems (Moscow, Russia). Manifestations of the “detachment” phenomenon in the crew – mission control center (MCC) communication previously identified in the Mars-500 project were confirmed in this study. As in the Mars-500 experiment, in the SIRIUS-19, the landing simulation in the halfway of isolation caused a temporary increase of crew communication with MCC. We also revealed several differences in the communication styles of male and female crew members. By the end of the experiment, there was a convergence of communication styles of all the SIRIUS crew members and also an increase in crew cohesion.

## Introduction

With the increase in the duration of the space missions, autonomy becomes one of the significant stress factors that need special attention. During the exploratory flight to Mars, communication delays will unavoidably diminish the effectiveness of the distant crew psychological support from the mission control center (MCC). While the autonomy factor gets combined with a variety of other stress factors (such as microgravity and sensory deprivation), crew members need to effectively cope with stressful conditions by themselves with limited autonomous resources available on board. At present, the strategy of selection of the cosmonauts for interplanetary flights remains uncertain and the search for measurable psychological indicators of effective coping with stress under isolation stays relevant.

The results of communication studies in the Mars-500 project (Ushakov et al., 2014) have shown that simulation of the autonomous spaceflight conditions, including the absence of additional resupplies and communication delay, may lead to manifestations of the psychological “detachment” phenomenon described by Kanas and Manzey (2008). According to the authors, it is caused by the progressive increase in crew autonomy with the loss of the visual image of their home planet that could negatively influence the mood, morale, and overall activity of the crew and induce groupthink (Janis, 1983).

Such a “detachment” may lead to resistance of crew members to the recommendations of the mission control and predominance of their decision-making based on their knowledge, values, and priorities. During the Mars-500 experiment, we observed a decrease in informing the crew of the MCC about their needs and problems and an increase in the number of independent decisions (based on knowledge, values, and goals of the isolated small group). Sometimes, this prevented the MCC from making effective decisions based on a clearer understanding of the situation on board and made it difficult to make practical recommendations. The increasing autonomy in the Mars-500 crew behavior was determined by the fact that after adaptation to isolation, the “marsonauts” realized that they learned how to solve emerging problems independently without any harm for the Mission Protocol. Analysis of communication data in the Mars-500 experiment (especially during and after the period of simulated “complete communication loss”) showed that the inability to receive confirmation of one opinion from the MCC immediately and, thus, to satisfy the need associated with the information insufficiency and coping with uncertainty, leads further to the mistrust and breaking of the established information circuit between the crew and the MCC. Both sides became more and more dissatisfied with these contacts, based on their further decisions not on the current data about the mutual positions, but the assumptions. We consider these processes in the interaction between the crew and the MCC as a manifestation of the “detachment” phenomenon, enhanced by increasing asthenization of the central nervous system due to isolation, monotony, confinement, lack of physical activity, and communication limitations (Myasnikov and Stepanova, 2000; Kanas and Manzey, 2008; Kanas, 2015).

The results of the Mars-500 experiment confirmed the concerns about a possible adverse effect of the communication delay on the quality of information exchange between the crew and the MCC. However, the small statistical sample on which these results were obtained required further research. That was made within the frame of the Scientific International Research in Unique terrestrial Station (SIRIUS) international project, consisting of several isolation studies of various duration (from 2 weeks to several months), which simulated a flight to the moon of the international mixed gender crews. In the SIRIUS project, the same set of pressurized chambers was used as in the Mars-500. During the experiment, the international crew simulates the execution of landing operations in the middle of the mission as in the Mars-500. Also, a 5-min delay in communication between the MCC and the crew is reproduced again and additional resupplies are rare. These operational similarities in the simulation of the interplanetary missions made it possible to compare the results of the psychological analysis of the communication of the crew, despite the different confinement duration.

The main difference in the Mars-500 and the SIRIUS experiments is the mixed gender crew. First studies on the mixed gender behavior and interactions of the crews under confinement were conducted in the Institute for Biomedical Problems (IBMPs) in the 1980s. During these studies, gender stereotypes led to interaction problems that created psychological tension and conflicts in the confined group (Orlov et al., 2018). The results of the experiments helped to organize proper medical and psychological support for the flights of the SE Savitskaya and EV Kondakova to the Soviet space stations. The Simulation Of Flight of International Crew on Space Station (SFINCSS-99) experiment, one of the first confinement experiments with mixed national and gender composition crew, simulated the International Space Station (ISS) crew interactions. One of the experimental crews (confined for 110 days) included a woman. As in the previous studies, crew members reported problems in interaction and one of the subjects left the chambers in the middle of the mission (Baranov et al., 2001). United States researchers also made studies involving gender-balanced male and female crews in the Flashline Mars Arctic Research Station (FMARS) program, simulation of human Mars mission. Studying the psychological adaptation peculiarities, Bishop et al. (2010) detected the differences in coping strategies used by the female and male crew members. They reported that for the male crew members, coping strategies of avoiding and ignoring problems were more typical. Female crews frequently demonstrated more effective coping, aimed to reduce emotional stress and resolve problems.

The research objective was to study the influence of various unfavorable factors of the interplanetary missions – long-term isolation, communication delay, stress, crew cohesion, and gender differences – on the crew communicative behavior. We also attempted to compare the crew communication data from the two longitudinal isolation studies simulating interplanetary missions, the Mars-500 and the SIRIUS, in order to define the general tendencies of isolated crew interaction with MCC under the communication delay.

## Materials and Methods

### Participants

A total of six volunteers took part in the 17-day isolation (SIRIUS-17): three women aged 28–37 years, all Russian, and three men aged 33–44 years: two Russian and one German.

The study in the SIRIUS-19 120-day isolation involved three women aged 29–33 years, all Russian, and three men aged 31–44 years: one Russian and two American.

Thus, in both the studies, the interaction between multicultural crews and national (Russian) MCC was observed.

### Bioethics and Informed Consent

The studies involving the human participants were reviewed and approved by the Bioethical Commission of the Institute of Biomedical Problems of the Russian Academy of Sciences (protocol No. 501 of February 18, 2019) and fully complied with the principles of the 1964 Declaration of Helsinki.

Each study participant voluntarily signed an informed consent after having the potential risks, benefits, and nature of the upcoming study explained to them.

### Design of the Study

The SIRIUS-17 and the SIRIUS-19, ground-based interplanetary flight chamber simulations, took place in the IBMP, Russia, from October to November 2017 and March to July 2019 accordingly. Both the experiments simulated operations and activities at the lunar orbital station: docking, remote control of the rover on the lunar surface, manipulations with a robotic arm, medical and psychological research, and maintenance operations in the pressurized chambers.

The SIRIUS-17 isolation was rather short to determine the longitudinal patterns of the crew communication; thus, the presented data were mainly obtained during the 120-day SIRIUS-19 experiment.

The 120-day long SIRIUS-19 mission included the following main stages: a flight to the moon followed by a flyby to search for a lunar landing site, a 6-day lunar landing of four crew members for the surface operations, stay on the lunar orbit, and remote control of the lunar rover to prepare the base, and then return to the base.

The main source of data for the analysis of communication between the crew and the MCC were daily (morning and evening) planning conferences [daily planning conference (DPC), similar as on board of the ISS] and other video messages recorded and sent by the crew. For ethical reasons, the analysis of private communication was not made.

The crew-MCC communication without signal delay (by phone) was implemented in the first and last 10 days of the 120-day isolation. On days 11–108, only delayed communication (5 min one way) was possible according to the Mission Scenario.

### Content Analysis Method

In the SIRIUS experiments, we used the traditional method of content analysis of crew communication with the MCC applied for inflight and ground experiments in the IBMP (Gushin et al., 2016). The content analysis method transforms the content of communication to a limited reproducible set of quantitatively measurable categories amenable to computer processing based on the clear coding rules (Krippendorf, 1980; Neuendorf, 2019; Yusupova et al., 2019). The main categories of our content analysis method represent the coping strategies. The idea of stress manifestation through the coping strategies was proposed by Lazarus and Folkman (1984), who demonstrated that the coping strategies are emotional, motivational, cognitive, and behavioral constructs that manifest in all the types of activities including speech. People use coping strategies to adapt to situations requiring psychological stability, to reduce the experienced stress level. One of the generally accepted classifications of the coping strategies places them on two independent scales (Lazarus and Folkman, 1984): problem-focused (problem solving) and emotion-focused (reducing one emotional stress) coping strategies. We should mention that one coping strategy may be problem focused or emotion focused in the different conditions (Scheid and Wright, 2017). In this study, we additionally used categories of the coping strategies described by Suedfeld et al. (2009) who was the first to make the content analysis of the memoires and interviews of the astronauts. More than 20 categories of analysis, including such important operational categories as “problem,” “demand/request,” “time” (a category related to the perception of time as a resource), and several categories reflecting the use of stress-coping strategies, used by the crew members to reduce stress and resolve the problem in communicative behavior, for example, “confrontation,” “avoiding,” and “taking responsibility” (Table 1).

**TABLE 1:** Coping strategies divided by communicative effectiveness.

Effective	Ambivalent	Ineffective
Trust Support Rational refuse Positive reappraisal Self-control Planning (planful problem-solving) Constructive initiative Taking/accepting responsibility Humor	Seek for support Subordination	Mistrust Confrontation Responsibility transfer Avoidance Sarcasm, irony

### Facial Expressions and Speech Acoustic Characteristics Analysis

Along with the content analysis, we used an automated analysis of video and audio recordings of the communication of the crew members with the MCC. One of the main indicators reflecting the functional state of the subjects is the general emotionality of non-verbal communication (facial expression). The opposite indicator is the share of neutral facial expressions, in other words, relatively unemotional, calm communication. The video recordings were processed by using the FaceReader software based on the validated (Stöckli et al., 2018) algorithm registering manifestations of basic emotions in human facial expressions. Several acoustic characteristics of a speech signal such as the fundamental frequency (F0), signal intensity (loudness), the number of voice pulses, shimmer, and jitter effects (variability of the speech signal in amplitude and frequency, respectively), correlating with the level of psychophysiological tension of the speaker, reflecting changes in his functional state were studied (Kartavenko, 2005; Frolov and Milovanova, 2009). The Praat software was used for the acoustic analysis of audio recordings.

### Additional Tests and Questionnaires

The State-Trait Anxiety Inventory (STAI) questionnaire was used to measure anxiety levels. To assess the interpersonal preferences in situations of work and leisure activities and cohesion, we used classical sociometry (Zhukov, 1981). We used the Personal Self-Perception and Attitudes (PSPAs) software (Vinokhodova and Gushin, 2014) for analysis of interpersonal perception – in particular, to assess how similar to themselves (or “close”) the subjects perceive their crewmates.

### Statistical Analysis

The used statistical methods included the ANOVA, factor analysis by the principal component extraction method (Varimax rotation method with Kaiser normalization), and comparison of the mean of two independent samples (the Mann–Whitney *U* test and the Wilcoxon rank-sum test).

## Results

### Dynamics of the Crew Communication Behavior During the Isolation

During the first 10 days of the SIRIUS-19 confinement, we registered highly intensive crew-MCC audio (phone) communication: 320 sessions of audio conversations with a total duration of 11 h. In the last 10 days of the isolation, when audio communication was possible again, we registered only 34 sessions of audio conversations with a total duration of 1 h 17 min.

After adapting to the experimental conditions (including communication delay that started from the 11th day of isolation), the crew, such as in previous long-term experiments (for example, the Mars-500), demonstrated autonomy and independence. During 4 months of isolation, the SIRIUS-19 crew transmitted 2,399 video messages to the MCC, including video of morning and evening DPCs from each crew member, of a total duration of 38 h 35 min. The MCC transmitted to the crew 1,047 video messages, including answers to questions asked during the DPC, with a total duration of 18 h and 40 min. During the isolation period, the crew also transmitted 145 text messages (“radiograms”) to the MCC and received almost 10 times more (1,035) radiograms from the MCC. Radiograms from the MCC, as a rule, included formal instructions on the methods performed during the corresponding days.

**FIGURE 1:**
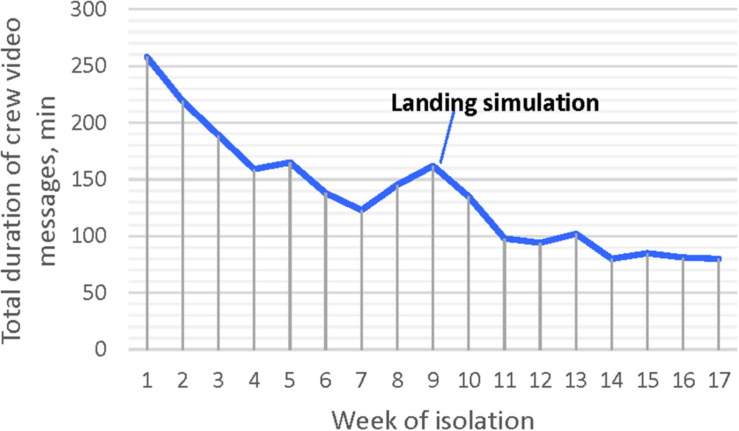
Video messages of the crew duration under isolation.

The number of video messages transmitted by the crew decreased during isolation – from 200 in the first week of isolation to 115–120 during the last weeks. The duration of video recordings also decreased significantly (Figure 1).

**FIGURE 2:**
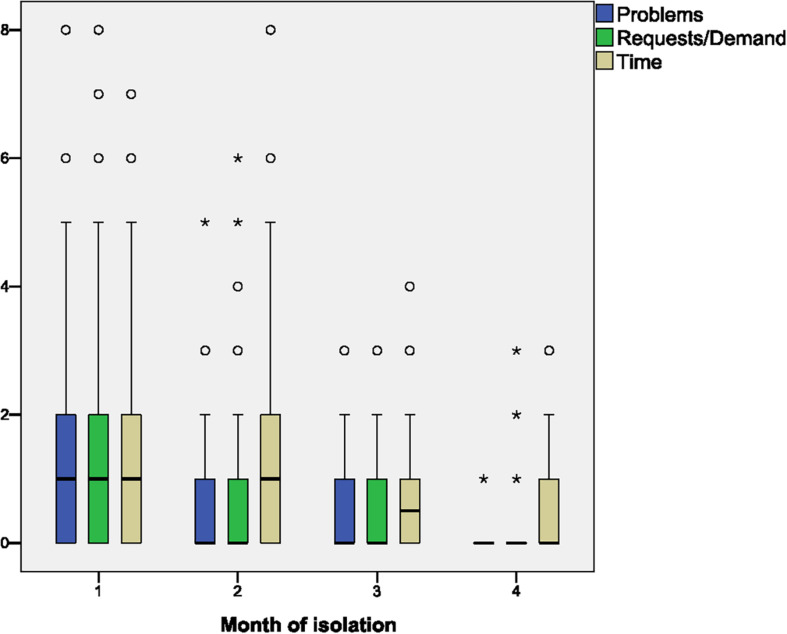
Monthly dynamics of the average daily number of statements in the “problems,” “requests,” and “time” categories in crew communication with the mission control center (MCC). Circles and asterisks indicate, respectively, outliers and extreme outliers (significantly different values on some days of the isolation, according to SPSS statistical calculations).

Content analysis of the audio and video communication showed a decrease in the expression of the needs and problems of the crew, seek for support. In particular, there was a significant decrease in the frequency of such categories as “problems,” “requirements/requests,” and “time” (Figure 2).

We also found a significant difference in the communication parameters in the first and the second halves of isolation. A temporary increase in external communication was observed only during simulation of the landing on the lunar surface (9–10 weeks of isolation).

The study of facial expressions in the video messages of the subjects during the 4-month isolation revealed significant changes in their emotional status (Table 2). For three crew members (B, C, and D), a decrease in the general emotionality of communication was observed, for two of the crew members (A and E) – an increase, i.e., subject A showed a significant increase in negative emotions with a decrease in positive ones, while subject E had the opposite tendency.

**TABLE 2:** Correlation of basic emotions expression in the facial expressions and isolation duration of the crew members.

Subject	Neutral	Joy	Sadness	Anger	Astonishment	Fear	Disgust
A	−0.467**	−0.168*	0.273**	0.187**	–0.051	0.542**	–0.021
B	0.328**	−0.181**	0.174*	–0.037	−0.272**	0.137*	0.063
C	0.586**	−0.136*	−0.375**	−0.667**	0.456**	−0.488**	−0.398**
D	0.275**	–0.014	–0.033	−0.418**	0.026	−0.282**	0.029
E	−0.470**	0.551**	−0.340**	−0.373**	0.240**	–0.080	−0.174*
F	0.022	0.020	–0.001	–0.069	–0.135	0.021	0.172*

*p < 0.05; **p < 0.01.

Analysis of the main acoustic indicators in crew talks with the MCC during the first month of the experiment confirmed the impact of the acute period of adaptation to the isolation conditions. During the first 4 days of isolation, the F0 and the voice volume (intensity of the speech signal) in all the subjects were higher than average. At the end of this period (by the fifth day), the acoustic parameters returned to the individual norms. Their next rise occurred on the 8–12th day of isolation when a 5-min communication delay began to influence communication. During this period, we observed an increase of F0 (significant), the intensity of speech signal, the number of voice impulses, and the proportion of non-sounded speech fragments.

**FIGURE 3:**
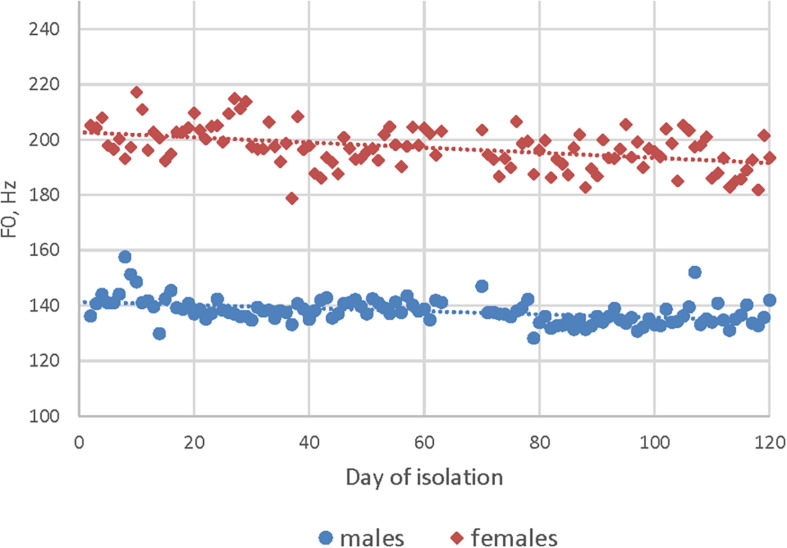
Dynamics of the fundamental frequency of speech in the Scientific International Research in Unique Terrestrial Station (SIRIUS) crew members during 120 days of isolation.

Study of speech acoustic characteristics revealed an increase in the number of voice impulses and shimmer effects in the speech signal during the isolation. Also, a decrease in the number of non-sounded speech fragments (pauses) and the fundamental frequency of speech was detected (Figure 3). Starting from the second month of isolation, a difference between the morning (lower) and evening (higher) F0 was noted for all the crew members, probably, corresponding to increasing fatigue level. The “moon landing” period was characterized by an increase in the number of pauses in speech, in F0 and speech volume, and in the number of voice impulses. At the same time, during this period, technical problems with speech recording often arose (due to the splitting of the crew in two parts for the “landing”) and, therefore, the obtained data were incomplete.

### Influence of Gender Differences on Communication Behavior Characteristics

In the SIRIUS-19 isolation, several differences in communication of gender subgroups were also revealed, while using verbal (content analysis) and non-verbal (speech acoustics and facial expressions) communication analysis methods.

According to the content analysis results, the statements of female crew members had an emotional connotation more often, both positive (*p* < 0.001) and negative (*p* < 0.001). Stress coping with the help of humor (*p* < 0.001) and positive reassessment of the situation (*p* < 0.001) also dominated among women. The informational component of communication was more pronounced in women due to the prevalence of such communication categories as initiative (*p* = 0.011), information (*p* = 0.002), and logistics (*p* = 0.021) compared to men. Female crew members were more likely to provide and seek support, they mentioned sleep quality (*p* < 0.001) more often, and also regularly talked about their efforts (*p* < 0.001) during the performance of working operations. In the speech of male crew members, the cognitive component was more pronounced (*p* < 0.001). We can also indicate a tendency among men to use confrontation as a way of social regulation in problem situations (*p* = 0.084).

**FIGURE 4:**
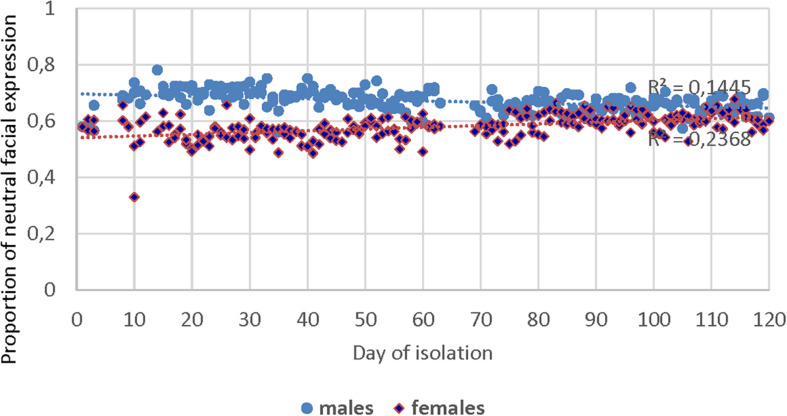
Dynamics of the share of neutral emotions in daily planning conferences during isolation.

The analysis of the communication videos of the crew in the SIRIUS-19 showed differences between gender subgroups in the manifestation of basic emotions (Table 3). In particular, in women, there were more manifestations of joy and sadness emotions. In contrast, men were more likely to demonstrate anger while discussing emerging issues. However, the study of emotional involvement level in communication according to the ratio of a calm facial expression (the so-called neutral expression) and positive/negative emotions showed the bidirectional trends: in men, the average proportion of neutral facial expressions decreased, while in women, this parameter increased during the isolation (Figure 4). Also, linear approximation reliability indicator values (*R*^2^ = 0.15 and *R*^2^ = 0.24 for men and women, respectively) do not allow to make an unambiguous conclusion about the statistical reliability of the observed linear trends.

**TABLE 3:** Comparison of gender subgroups by the basic emotions.

Significance criteria	Neutral	Joy	Sadness	Anger	Astonishment	Fear	Disgust
Mann–Whitney’s *U*	54	13	17	50	46.5	58.5	72
Wilcoxon’s *W*	132	91	95	128	124.5	136.5	150
*Z*	−1.039	−3.421	−3.2	−1.384	−1.935	−1.2	0
Asymptotic significance (two-tailed)	0.299	0.001	0.001	0.166	0.053	0.23	1
Exact significance	0.319	0.000*	0.001*	0.219	0.143	0.443	1.000

*p < 0.05.

**FIGURE 5:**
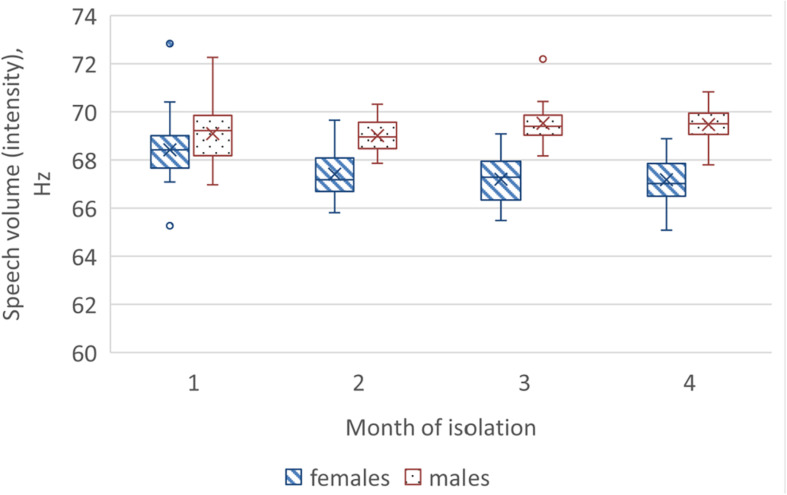
Dynamics of speech volume in female and male crew members during isolation.

**FIGURE 6:**
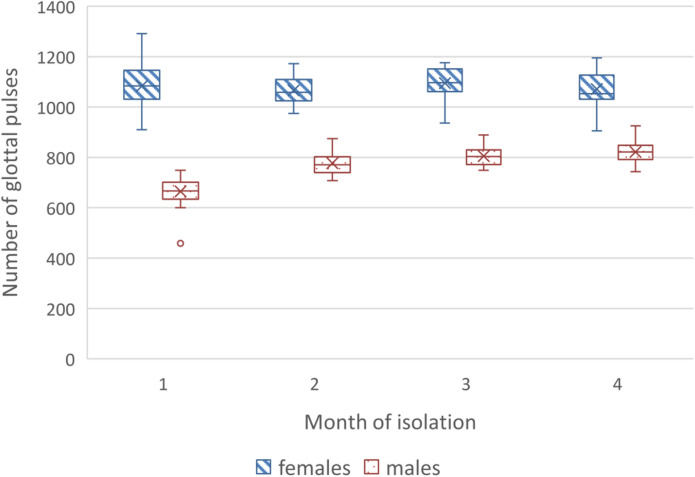
Dynamics of vocal (glottal) pulses in female and male crew members speech.

In the 4-month isolation experiment, we also found differences in dynamics of acoustic speech indicators between the gender subgroups of the SIRIUS-19 crew. In women, we noted a decrease in speech loudness by the end of the experiment (Figure 5), while in men, we observed an increase in the number of vocal pulses (Figure 6). The number of non-sounded speech fragments (pauses) in the speech of the men was higher than in the speech of the women during the first month of the experiment. The difference in indicators that were recorded during the stressful periods of isolation (including the acute period of adaptation) and in “normal” (stable) experimental conditions was less pronounced in women than in men. The differences between male and female subgroups in the number of acoustic indicators (voice impulses and non-sounded speech fragments) decreased during isolation (as shown in Figure 6).

### Communication Parameters Convergence and Crew Cohesion During the Isolation

It should be generally noted that while the male and female parts of the SIRIUS-19 crew showed significant differences in the style and content of their communication with the control center in the first month of isolation; then, during the experiment, these differences were smoothed out (Figures 4, 6).

It is quite interesting that similar tendencies were also noted according to the data of sociopsychological studies. In the course of isolation, the sociometric cohesion increased and the structure of the group consolidated for the situation associated with participation in the isolation experiment (work activity). The sociometric structure according to the criterion of “joint leisure activities” from the very beginning reflected a very high level of cohesion of the crew (Table 4).

**TABLE 4:** Crew cohesion criteria during the 4-month isolation.

	Day of isolation					
	18	36	76	91	103	116
Criterion 1 (work activity)	0.56	0.67	0.67	0.67	0.67	0.67
Criterion 2 (leisure activities)	0.78	0.78	0.56	0.56	0.56	0.56

Only after separation of the crew during simulation of the landing, cohesion for the second criterion significantly decreased (*p* = 0.042) and in the last month, they arise tendencies toward less consolidation and the appearance of isolated group members.

**FIGURE 7:**
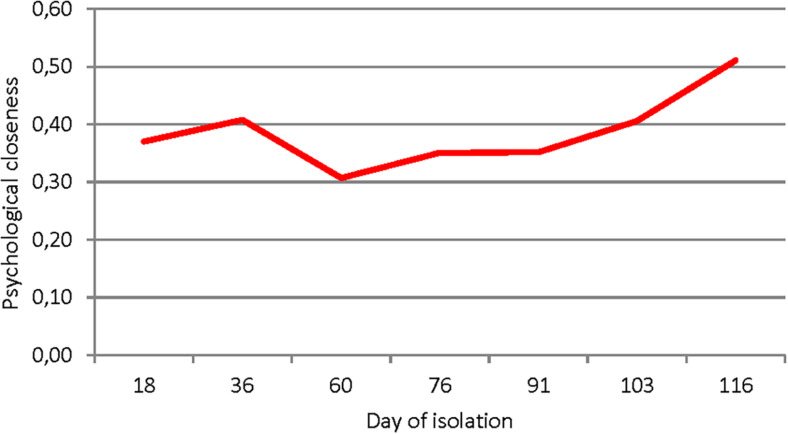
The psychological closeness of the subjects (whole crew) in the course of isolation.

There was an increase in psychological similarity or “closeness” during isolation according to the PSPA results (Figure 7). In this technique, “distances” are measured between the “real self” of each subject and the perceived images of other crew members in the personal psychosemantic space. Psychological “closeness” is calculated as the reciprocal of distance: the greater the distance, the less closeness, and vice versa. During the experiment, the subjects began to describe other crew members as more similar to themselves. These results are consistent with sociometric data about group cohesion for “work” situations.

### Coping Strategies and Levels of Reactive Anxiety

The respondents with a medium level of reactive anxiety were 2.24 times more likely to use “informing” statements and used “self-control” statements three times more often than those with low anxiety levels. Also, subjects with medium anxiety levels did not use the “constructive initiative,” “claims,” and “requests” strategies at all (according to content analysis data). These latter three categories were used only by the participants in the low anxiety level group (Table 5). It should be noted that the candidate selection methodology used for isolation experiments, same as the cosmonaut selection procedures, reduces the probability for highly anxious candidates to be accepted to the crew. Due to that, the SIRIUS participants showed medium and low (but never high) scores on Spielberger’s reactive anxiety rating scale.

**TABLE 5:** Differences in use of content analysis categories between the subjects with low and average reactive anxiety levels.

Categories	Low level of reactive anxiety (average values + SD)	Medium level of reactive anxiety (average values + SD)	Mann–Whitney *U*	Significance
Informing (sharing information)	15.47 ± 6.88	34.67 ± 16.22	5	0.037
Self-control	1.56 ± 0.74	4.67 ± 2.22	2.5	0.014
Requests, demands	3.2 ± 1.76	0	0	0.007
Criticism	4.73 ± 3.16	0	6	0.043
Initiative (constructive)	3.27 ± 3.17	0	7.5	0.06

## Discussion

In space flights, crew communication with the MCC is not only providing the crew with the necessary operational data but also gives additional external stimulation to prevent sensory and social deprivation (Kanas, 2015; Yusupova et al., 2019).

So, on one hand, a decrease in communication volume reflects the better adaptation of the subjects to long-term isolation simulating unfavorable conditions of space flight. Therefore, we interpret the decrease of communication volume as the diminishing need for external stimuli *via* the audio and video contacts with the MCC to withstand the sensory and social deprivation and monotony. On the other hand, the decrease of communication through the isolation period is associated with progressive mastering of skills needed for the execution of Mission Protocol and equipment management. That helps to gain relative independence from the MCC information support. In comparison with real space flight, under isolation, the crew is doing more standard operations and less involved in technical maintenance of the “spaces station,” so they less need permanent input from the specialists. A decrease in the number of statements, primarily related to the planning and organization, such as “needs” and “problems” of life and work in the chambers, may also indicate further adaptation to the conditions of the isolation and increase in the operational independence of the crew.

It is even more important that crew communication tendencies were similar to those observed in the Mars-500 (Ushakov et al., 2014). As in the previous simulation of an interplanetary mission, international crew decreased communication volume throughout the isolation period. Also, such as in the Mars-500, video messages turned to be the main communication channel of the SIRIUS mission, because, according to the interviews of the crew members, it took less time than written radiograms and allowed to make contacts less formal. In both the experiments, the most important event, landing simulation, caused a significant temporary increase in the crew-MCC contacts. Finally, as in the Mars-500, the crew did not demonstrate benevolence in contact with the MCC, less sharing with earth their problems, and needs in the second part of the mission.

It can be assumed that the consistent decrease in communicative activity is a reflection of the processes of autonomization and possibly mental asthenization of the crew, the effects that were observed in several previous studies (Myasnikov and Stepanova, 2000; Sandoval et al., 2012). Another possible explanation described in the literature and partly confirmed in the “content” space experiment (Russian psychological study of ISS communication of the crews with the MCC) could be a so-called “third quarter” phenomenon (Kanas, 2015; Yusupova et al., 2019). Further studies are needed to additionally test this hypothesis.

The data about gender differences influence obtained in the SIRIUS confirm some of the results of Bishop et al. (2010) from the 4-month “Martian” space simulation experiment focused on differences in the coping strategies used by men and women. However, the study of stress impact on the facial expression and voice acoustics of the subjects mostly demonstrated the individual differences. Also, it should be noted that all the female subjects were Russian, while men were Russian, German, and American, so the cultural background could influence the observed differences.

For the first time convergence in communication, the behavior of subjects with different cultural backgrounds and genders was detected during the isolation period. Also, the increase in crew cohesion was detected simultaneously. It can be assumed that, as in the autonomous long-term international expeditions of the famous traveler Thor Heyerdahl, difficult conditions of isolation experiments contributed to the convergence of life positions and also styles of behavior and communication of the crew members (Senkevich, 2017).

A study previously conducted in the Mars-500 isolation experiment (Kuznetsova et al., 2017) showed a correlation between the state anxiety and the capacity for emotional self-regulation (as an aspect of psychological stress resistance). Crew members with low state anxiety showed higher results in positive emotional self-regulation that was developing throughout the experiment, while crew members with higher anxiety results showed an opposite trend.

To analyze the differences in communicative strategies between the subjects with low and medium anxiety levels, we also turned to the psychodynamic theory, quite esteemed in psychotherapeutic and clinical practice, and transactional theory of communication by Berne (1961). He identified “horizontal” (on equal terms, in his terminology – “adult-adult”) and “vertical” (situations of inequality, “older–younger” or “parent–child”) types of communicative interactions. When applying Berne’s theory to the results of this study, we can see that the tendency to “horizontal” (equal) interaction as equal cooperation was observed in the participants with low anxiety levels and manifested itself in the use of problem-focused coping strategies, as “constructive initiative,” “informing,” “requests and demands,” and “claims.” The tendency to “vertical” (unequal) interaction was observed in the participants with medium anxiety levels and manifested itself in the use of a “self-control” coping strategy, mainly focused on suppressing emotional stress.

## Conclusion

Analysis of the communication of the international mixed gender crews in the SIRIUS experiments confirmed manifestations of the “detachment” phenomenon in the crew–MCC communication previously identified in the Mars-500 project. When crew members adapted to the mission conditions, they needed less recommendations of the MCC and this manifested both in a decrease in the total communication volume and in the expression of information needs that the MCC could satisfy in conditions of communication delay. As in the Mars-500 experiment, in the SIRIUS-19, the landing simulation in the halfway of isolation caused a temporary increase of crew communication with the MCC.

We also revealed some differences in the communication styles of male and female crew members, confirming the results of Bishop et al. (2010) from the Martian simulations. The subgroup of women more often (than the subgroup of men) informed the MCC about existing problems, but also regularly expressed their support and their communication was, in general, more emotional. Despite the initially revealed differences in communication both in the gender subgroups and in individuals, by the end of the experiment, there was a convergence of communication styles of all the SIRIUS crew members in such parameters as communication volume, its emotionality, and certain acoustic indicators. However, it is too early to make more general conclusions due to small sample sizes (six men and six women) and pronounced individual differences in all the subjects.

## Data Availability Statement

The raw data supporting the conclusions of this article will be made available by the authors, without undue reservation.

## Ethics Statement

The studies involving human participants were reviewed and approved by the Bioethical Commission of the Institute of Biomedical Problems of the Russian Academy of Sciences. The patients/participants provided their written informed consent to participate in this study.

## Author Contributions

All authors listed have made a substantial, direct and intellectual contribution to the work, and approved it for publication.

## Conflict of Interest

The authors declare that the research was conducted in the absence of any commercial or financial relationships that could be construed as a potential conflict of interest.

## Publisher’s Note

All claims expressed in this article are solely those of the authors and do not necessarily represent those of their affiliated organizations, or those of the publisher, the editors and the reviewers. Any product that may be evaluated in this article, or claim that may be made by its manufacturer, is not guaranteed or endorsed by the publisher.
